# Marker Peptides for Indicating the Spoilage of Milk—Sample Preparation and Chemometric Approaches for Yielding Potential Peptides in a Raw Milk Model

**DOI:** 10.3390/foods13203315

**Published:** 2024-10-18

**Authors:** Lisa-Carina Class, Gesine Kuhnen, Jasmin Schmid, Sascha Rohn, Jürgen Kuballa

**Affiliations:** 1 GALAB Laboratories GmbH, Am Schleusengraben 7, 21029 Hamburg, Germany; lisa-carina.class@galab.de (L.-C.C.); gesine.kuhnen@galab.de (G.K.);; 2 Hamburg School of Food Science, Institute of Food Chemistry, University of Hamburg, Grindelallee 117, 20146 Hamburg, Germany; 3 Department of Food Chemistry and Analysis, Institute of Food Technology and Food Chemistry, Technische Universität Berlin, Gustav-Meyer-Allee 25, 13355 Berlin, Germany; rohn@tu-berlin.de

**Keywords:** raw milk, spoilage, proteomics, mass spectrometry, Python, chemometrics

## Abstract

The diminishing of food waste is gaining increasing importance, especially in context with a growing population and a need for the sustainable use of food resources. A more precise determination of the best-before date can contribute to this general aim. As proteoforms can be regarded as indicators for ecophysiological influences, their suitability for determining the spoilage and, consequently, the shelf-life of food is suggested. Proteoforms reflect the spoilage of food more accurately. The aim of the present study was to develop an efficient proteomics workflow to determine the shelf-life of milk as a prominent target. In this case, raw milk was chosen as model, as it degrades much faster. The integration of different multivariate analysis techniques was used to analyze the spoilage of raw milk with regard to aspects of its proteome. As the feasibility of such an approach has already been demonstrated in previous studies, it is further necessary to enable a robust and reproducible workflow, primarily gaining appropriate numbers and amounts of peptides when the research question differs and other dairy products are evaluated. In the present study, two approaches for gaining peptides were considered: In addition to a direct hydrolysis of a protein-rich sample solution, in-gel hydrolysis is another common approach in proteomics. By separating the proteins in a traditional gel electrophoresis before hydrolysis, the change in the individual proteins and, consequently, potential peptides can be monitored more specifically during storage. However, the traditional approach offers not only possibilities but also limitations that must be considered. The study showed that it is beneficial to apply a combination of different application strategies, as they complement each other and can thus increase the information content of a sample or confirm a theory. Mass spectrometric features, which represent a chemical–structural change of all kinds of compounds during storage, were selected, and three of them were identified as peptides, originating from α-s1-casein.

## 1. Introduction

Peptide markers are common targets for monitoring the transformation of protein-rich foods and ingredients. To name but a few, peptide markers can be used, e.g., to determine meat authenticity [1], to estimate the whey content in cheese [2], to detect allergens [3], or to evaluate seafood quality [4]. Furthermore, peptide markers can be used to determine the shelf-life, as the proteome reflects ecophysiological influences during the storing of a product [5].

Shelf-life of food, based on food spoilage, is again an increasingly field of interest, as nowadays sustainability and socioeconomic aspects need to be considered additionally [6,7,8,9]. However, food waste statistics show that around 50% of the most common waste along the value chain is assigned to private households [10,11,12]. This is partly due to the fact that the (food safety) meaning and the labelling of the best-before date are generally not fully clear to the average consumer [12,13,14]. Unfortunately, it is common practice to dispose of food immediately after passing the best-before date, as it is too often regarded as expiry date, although the product is in most cases still suitable for consumption [9]. The average consumer rates food less positively after the best-before date and is more likely to throw it away without checking the actual quality, e.g., by terms of a simple sensory evaluation of the smell of the product [15,16]. In the original food production facilities, sensory and microbiological methods are regularly used to estimate a potential shelf-life [11,14,17,18,19]. Unfortunately, these methods are often not suitable for predicting the “real” expiry date of food [9]. The best-before date is specified by manufacturers with a safety margin for avoiding any risk to consumer’s health [9]. Economic problems occur when a company does not use a safety margin. When there is a customer complaint or, even more severe, a recall of a food product, the company’s reputation can suffer, and the number of sales can decrease as a result. The development of easy and fast analytical workflows that enable a coherent and valid analysis of the spoilage of food and, thereby, help to improve the shelf-life estimation can be a chance to minimize food waste. As already mentioned, the continuous release and formation of certain peptides or peptide profiles can be used to monitor spoilage. All external conditions and environmental influences have an effect on the quality of milk, especially on raw milk. The most prominent results are varying microbial population structures (in milk) [20]. Psychrotrophic bacteria can accelerate the spoilage of milk and dairy products even when the dairy products are sterilized or otherwise treated. Heat-resistant enzymes (e.g., peptidases and lipases) especially can escape from heat treatments [20]. As a consequence, heat-resistant proteases are able to degrade the milk proteins and thus continuously produce endogenous peptides [21]. However, this can be useful for characterizing a wide range of external influence factors, not only in milk and dairy products but further in all food with a significant protein content. Peptide spoilage markers were used, for instance, in a study described by Wei et al. [22]. For milk, Dalabasmaz et al. also analyzed the peptide profile for identifying storage markers, mainly considering peptides produced by milk proteases or bacterial proteases, which are endogenously present in the milk [23]. Milk and milk products are an important group of food for shelf-life and spoilage analysis, as products can differ significantly from raw to highly processed and microbiological spoilage occurs frequently and also with large variation.

However, there are only a limited number of studies that investigated the changes in the proteome of milk over time towards spoilage [24,25,26].

Marker peptides can be obtained by different approaches. Most prominently and still developing quite fast, the mass spectrometric analysis of proteins is an established tool (‘proteomics’) [27]. However, there is certain variability when considering sample preparation options and data evaluation possibilities. The most simple and obvious approach is to initially analyze the free peptides that develop over time when being released in protein-rich foods by endogenous enzymes or microbial degradation, such as under (food) storing conditions [23].

A more strategic and well-established approach in proteomics is to analyze peptides after an artificial degradation with an enzyme that leads to a significant number of peptides. The approach of analyzing (all) peptides released by artificial but reproducible degradation/digestion processes enables us to analyze the changes in the proteome rather than only individual peptides and ensures adaptivity to future studies. There are two ways to follow this so-called bottom-up strategy. The first one is “in-gel hydrolysis”, where the individual proteins are separated prior to enzymatic hydrolysis [28]. The advantage of this method is particularly noticeable in the interpretation of the data, as proteins can be separated and assigned to a certain position in the gel. Afterwards the potential marker peptide can then be related to the original protein. The main issue, however, is the multiple small sample preparation steps that are time-consuming and vulnerable to contamination. The second approach is known as “in solution hydrolysis”, in which the (food) sample of interest (in this case, milk) is directly hydrolyzed enzymatically without the proteins being separated previously [29,30]. This is an approach that is by far more time-efficient but more complex for identifying and assigning proteins or marker peptides, as the number of signals is significantly higher because it involves the whole proteome.

In a previous study, an initial proteomic workflow has been developed that was able to monitor the change of the milk proteome after heat exposure (‘pasteurization’). That approach was a bottom-up proteomic workflow with in-solution hydrolysis [5,31]. However, heat treatment and storing, especially excessive storing that leads to a protein decomposition by different endogenous enzymes (e.g., cathepsins) or microbial degradation, might lead to different protein and peptide profiles. Descriptions in the literature indicate that a degradation caused by enzymes that naturally occur in the raw milk, like plasmin, cathepsins, and elastase, change the proteome continuously over time [32]. Consequently, the workflow developed previously must be checked for appropriateness and robustness. When food spoilage leads to a dominant influence of countless microorganisms, the number of proteins involved becomes higher and higher, accordingly. This implies that also the sample preparation as well as the data evaluation need to be reconsidered and optimized.

The aim of the present study was to optimize a holistic method for selecting and identifying marker peptides for enabling an accurate evaluation of milk’s spoilage. These marker peptides could then link this approach to the estimation of the shelf-life in future studies. For this purpose, raw milk was stored over a period of ten days and analyzed. In this study, raw milk was exemplarily chosen for a method development, as it spoils in rather short time. As recently established for thermally treated milk, measurement was carried out using liquid chromatography–electrospray ionization–ion mobility spectrometry–quadrupole time of flight (LC-ESI-IMS-QToF) [5]. For the sample preparation, in-gel hydrolysis and in-solution hydrolysis were compared in order to neither lose information (when being limited by in-gel hydrolysis) nor to complicate data evaluation because of too much interfering proteins/peptides, as in samples that already begin to spoil. Obviously, milk’s shelf-life is a time-dependent process that should be adequately captured by data evaluation. Therefore, a partial least square regression (PLSR) step was added as an essential part in the data evaluation. All tools used to perform the computational data analysis were executed in the Python ecosystem [31]. The innovation of the presented study is the combination of different established preparation methods for protein hydrolysis and selected chemometric methods.

## 2. Materials and Methods

### 2.1. Reagents and Chemicals

HPLC-grade water, HPLC-grade acetonitrile, and isopropanol were purchased from VWR International GmbH (Darmstadt, Germany). Formic acid and hydrochloric acid were obtained from Biosolve B.V. (Valkenswaard, The Netherlands), while dithiothreitol (DTT), acetic acid, iodoacetamide (IAA), urea, calcium chloride dihydrate, and sodium bicarbonate were purchased from Sigma-Aldrich Chemie GmbH (Schnelldorf, Germany). Ammoniumhydrogencarbonate was obtained from Thermo Fisher Scientific Inc. (Waltham, MA, USA). Acrylamide, ammonium peroxodisulfate, bisacrylamide, bromphenol blue, glycerol, glycine, sodium dodecyl sulfate (SDS), tetramethylethylenediamine, and tris-hydrochloric acid were purchased from Carl Roth GmbH & Co. KG (Karlsruhe, Germany). Methanol was obtained from Th. Geyer GmbH & Co. KG (Renningen, Germany). The lock mass leucine enkephalin and the calibration standard for the LC-ESI-IMS-QToF analysis were purchased from Waters Corp. (Milford, MA, USA).

### 2.2. Proteins

As model proteins, the six most abundant proteins in milk were chosen. α-Lactalbumin (≥90% purity) was purchased from US Biological Inc. (Salem, MA, USA). All other proteins comprising β-lactoglobulin (≥90% purity), bovine serum albumin (BSA) (≥98% purity), α-casein (≥70% purity), β-casein (≥98% purity), and κ-casein (≥70% purity) were obtained from Sigma-Aldrich Chemie GmbH (Schnelldorf, Germany). Trypsin was from porcine pancreas with a specific activity of 5000 usp-u/mg protein and purchased from Carl Roth GmbH & Co. KG (Karlsruhe, Germany). As molecular weight markers, the PageRuler^TM^ Prestained Protein Ladder, 10 kDa to 180 kDa, obtained from Thermo Fisher Scientific Inc. (Waltham, MA, USA) was used.

### 2.3. Sample Material

All milk samples were purchased from a local farm, “Milchhof Reitbrook” located in Hamburg, Germany. The original milk samples were not pasteurized and homogenized but cooled directly after milking the cows. The samples were purchased on the day of the milking, and the study was initiated on the same day that the milk was produced.

### 2.4. Sample Preparation and Extraction

#### 2.4.1. Study Design

**Figure 1:**
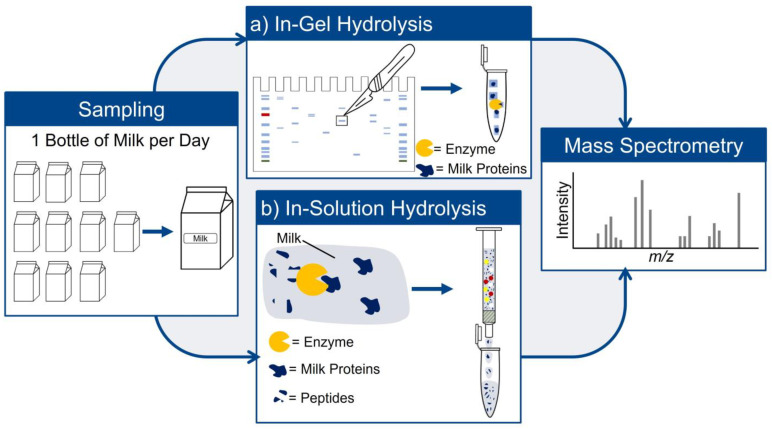
Schematic study design.

To investigate the behavior of the proteome during storage, ten bottles of raw milk were purchased and stored at 4 °C. Samples were taken for ten days. Every day, a fresh milk container was opened and used as sample material for sample preparation by gel electrophoresis and enzymatic in-gel hydrolysis and enzymatic hydrolysis in-solution without prior separation. Figure 1 shows the schematic design of the study.

#### 2.4.2. Sample Preparation

The first preparation of the samples was performed with SDS-polyacrylamide gel electrophoresis (PAGE). A ratio of 1:50 (*v*/*v*) dilution of the milk samples with water was performed to prevent overloading the gels. For the enzymatic hydrolysis without prior separation on gel, the milk was diluted according to Giansanti et al., (2016) so that the total protein concentration per individual sample was 90 µg [33].

#### 2.4.3. SDS-PAGE Analysis

For the electrophoretic separation, one dimensional discontinuous-SDS-PAGE was performed using a typical standard operating procedure. Briefly, analysis was performed using an 18% polyacrylamide gel as well as a 5% stacking gel of 1.0 mm thickness. An amount of 15 µL of the 1:50 (*v*/*v*) diluted milk solutions was mixed with 15 µL of a twofold concentrated sample buffer. This sample buffer contained tris-hydrochloric acid (pH 6.8), 2% SDS, 25% glycerol, 0.01% bromphenol blue, and 1.6% DTT. The solution was incubated at 95 °C for 5 min. Afterwards, samples were centrifuged (2000× *g*) and cooled down to 25 °C. Subsequently, 20 µL of the liquid samples were loaded onto the gel. In addition, 5 µL of a molecular weight marker was applied on a separate lane on each gel. The electrophoresis was performed with 100 V, 300 mA, and 10 W for 15 min, followed by 150 V, 300 mA, and 10 W for 100 min and the last electrophoresis step with 300 V, 700 mA, and 150 W for 15 min.

To visualize the protein bands, gels were stained for two hours with 0.025% Coomassie Brilliant Blue R250 in 40% methanol in water, containing 7% acetic acid. A washing step was then performed to remove excess color. For this, a decolorizing solution (50% water, 40% methanol, 10% acetic acid) was applied for 30 min at 25 °C.

#### 2.4.4. Enzymatic In-Gel Hydrolysis

The in-gel hydrolysis was performed based on a protocol described by Shevchenko et al. [28,34]. For tryptic in-gel hydrolysis, the most visible bands (Nos. 2, 5, 6, 8–11) from each lane, which resemble a sample day, were excised from the SDS-PAGE gels (1 mm × 1 mm cubes) with a scalpel, destained in different washing steps (I: water, II: ammonium hydrogen carbonate buffer (100 mM), and III: 50% acetonitrile). After the pieces of gel were destained, they were dehydrated under vacuum to dryness. After that, they were rehydrated with a reduction buffer containing 10 mM DTT, followed by an alkylation step with a 55 mM IAA solution. The pieces were then again dehydrated to dryness.

For the enzymatic hydrolysis, all solutions used were cooled to 4 °C. The digestion buffer containing trypsin, water, and ammonium hydrogen carbonate buffer (100 mM, 50/50/15, *v*/*v*/*v*) were added to the dry gel pieces until they were completely rehydrated. An incubation buffer (water, 100 mM ammonium hydrogen carbonate, and 120 mM calcium chloride solution) was added until the gel pieces were completely covered. Thereafter, the gel pieces were incubated at 37 °C for 16 h.

To extract the peptides from the gel pieces, an extraction buffer (25 mM ammonium hydrogen carbonate) was added and incubated at 37 °C for 15 min. Reaction was stopped by adding formic acid (5%, *v*/*v*). After an incubation time of 15 min at 37 °C, the solution was concentrated to dryness and redissolved in 300 µL of 0.1% (*v*/*v*) formic acid.

#### 2.4.5. Enzymatic Hydrolysis of the Whole Milk (“In-Solution” Hydrolysis)

The enzymatic in-solution hydrolysis of the samples was performed according to Giansanti et al. with the protease trypsin [33]. Therefore, milk samples were concentrated until dryness and redissolved in 2 M urea in water. The incubation time of the enzymatic hydrolysis was 12 h at 37 °C. Last step of the sample preparation was accomplished with a solid phase extraction (SPE) with Sep-Pak^®^ C18 cartridges (Waters GmbH, Eschborn, Germany) for purification [33]. The cartridges were conditioned with 100% acetonitrile, followed by an equilibration step with 0.6% acetic acid in water. After applying the sample solutions, a washing step with 0.6% acetic acid was carried out. Subsequently, the elution step to obtain the peptides was performed with aqueous 80% acetonitrile (0.6% acetic acid). The peptide solutions were prepared for the mass spectrometric measurement by concentrating to dryness and redissolving in 500 µL of 0.1% formic acid in water. Five replicates for each sample preparation were analyzed.

### 2.5. UPLC-IMS-QToF Analysis

The samples were analyzed with an ACQUITY^®^ UPLC I-Class (ultrahigh performance liquid chromatography) system coupled with a Vion IMS-QToF-MS (ion mobility spectroscopy quadrupole-time-of-flight mass spectrometer) (all Waters Corp., Milford, MA, USA).

The chromatographic separation was performed with an ACQUITY^®^ UPLC BEH C8 column (150 mm × 2.1 mm, 1.7 µm, 130 Å; Waters Corp., Milford, MA, USA) at 40 °C and a flow of 0.2 mL/min. Water with 0.1% formic acid was used as mobile phase A. Mobile phase B was acetonitrile with 0.1% formic acid. The gradient was set as follows: 0.0 min (99% A), 1.0 (99% A), 10.0 min (58% A), 12.0 min (15% A), 15.0 min (15% A), 16.5 min (99% A), and 19.5 (99% A). An amount of 2 µL of the sample extracts were injected. The autosampler was set to 10 °C.

For the detection of the analytes, the mass range was set to *m*/*z* (mass-to-charge ratio) 50–2000. Positive ion mode was used. Source temperature was set to 120 °C, and desolvation temperature was set to 450 °C. Cone gas flow was 50 L/h, and desolvation gas flow was 800 L/h (both nitrogen). The capillary voltage was set to 0.50 kV, the sample cone voltage was set to 40 V, and the source offset voltage was set to 80 V.

The mass spectrometric device was an MS^E^-instrument, producing simultaneously low- and high-energy spectra. An amount of 6 eV was used as collision energy to obtain the low-energy spectra. The high-energy spectra were obtained by using a ramp with elevated collision energy. The ramp was set from 15 eV to 45 eV.

### 2.6. Data Conversion and Computational Framework

The acquired raw data was transformed from the producer-specific uep-format to the universal mzML-format with MSConvert (ProteoWizard, Version 3.0.20340) [35].

Data analysis was performed using the programming language Python (Version 3.9.16) [36] and the development environments JupyterNotebook (Version 6.5.3) and PyCharm Community (Version 2020.3.3). Python packages as follows were used: pyOpenMS (Version 2.7.0), pandas (Version 1.5.3), Scikit-Learn (Version 1.2.2), numpy (Version 1.24.2), matplotlib (Version 3.7.2), and seaborn (Version 0.12.2).

### 2.7. Data Analysis of the In-Solution Hydrolysis

The preprocessing of the data and the feature extraction was performed similar to the approach described in a previous study [31]. Features are signals caused by all kinds of analytes in an analytical system. The use of this expression is very common in mass spectrometric-based bioinformatics. Therefore, features are initially not equal to peptides but can represent also other signal-responsible compounds besides peptides.

The mass spectrometric data were read as mzML-files with pyOpenMS. The data were centroided, and features were extracted with the FeatureFinderCentroided function from pyOpenMS. The features were saved in two pandas.DataFrames. One data frame contained the intensities of the features in the samples. The other data frame contained further information about each feature. It contained the *m*/*z*, the retention time in seconds, and the estimated charge of the features. Similar features were merged into one feature with an absolute tolerance in the *m*/*z* of 0.01 and absolute tolerance in the retention time of 2 s. The resulting data frame was used for analysis with a principal component analysis (PCA), partial least square discriminant analysis (PLS-DA), and PLSR.

PCA was performed with scikit-learn. The components were set to two. As it was used for a first look on the data, neither feature selection nor further processing proceeded in advance of the PCA; only the data were scaled with the function MinMaxScaler from scikit-learn.

PLS-DA was performed with two components. Features were selected by their variable importance in projection (VIP), and VIP scores were calculated for each feature. Only features with a VIP score higher than 1.5 were kept and used for the PLS-DA model. This model was validated, splitting the dataset into a training set (80%, 40 samples) and a test set (20%, 10 samples). The train_test_split function from scikit-learn was used for the split. The PLS-DA was built with the function PLSRegression and a LabelBinarizer (both scikit-learn).

The features for the data analysis were selected based on the F-value. This was performed with the SelectKBest function from scikit-learn [37]. The select number k was set to 1000. With these 1000 features, the PLSR was performed. The dataset was split into a train set (80%, 40 samples) and a test set (20%, 10 samples). For the split, the train_test_split function from scikit-learn was used with a random state set to 42. The regression was performed using the PLSRegression function from scikit-learn. The number of components was selected by the best value for R^2^ and the mean squared error (MSE). Figure S1 (Supplementary Materials) shows the MSE and R^2^ depending on the selected number of components. Based on this, with two components, the analysis proceeded. To select the feature with the biggest influence on the model, the VIP score of each feature was calculated.

### 2.8. Data Analysis of the In-Gel Hydrolysis

Two data frames of this experiment were generated in the same manner as described for the in-solution hydrolysis experiment. Features that were also detected in the blank samples were eliminated from the data frame. This elimination was performed with the same tolerance as the merge (*m*/*z* tolerance: 0.01, retention time tolerance: 2 s).

For data analysis, the data frame was separated based on the SDS-PAGE band that it was acquired from. With that, a data frame from each band was acquired. PLSR (PLSRegression function from scikit-learn) was performed with each of these data frames, and the number of components was set to two. Due to the limited amount of data, no validation was conducted. Tolerance for the search of a feature from the in-solution hydrolysis in the in-gel hydrolysis samples was set to *m*/*z* 0.01 and 5 s (absolute).

### 2.9. Identification of the Separated Protein Bands

The mass data collected during the UPLC-ESI-IMS-QToF analysis were converted from the device specific uep-files (unifi export package) to mgf-files (mascot generic file) and afterwards interpreted using MASCOT (Matrix Science, London, UK). The MASCOT search was performed against SwissProt database. The search parameters were the enzyme trypsin, peptide charge (1+, 2+, 3+), unrestricted protein mass, peptide mass tolerance ± 1.2 Da, fragment mass tolerance ± 0.6 Da, and the monoisotopic mass value. Further, one missed cleavage and variable modifications, as, e.g., carbamidomethyl, were allowed in the search. Specific isoelectric point or molecular weight restrictions were not considered. To validate the significance of the identified match, MASCOT uses a scoring system based on the probability. The values are calculated, and if the values are above 70, these matches can be considered significant.

### 2.10. Identification of Peptides

The identification of peptides proceeded as described by Kuhnen et al. [31]. The changes to the workflow are described as follows. The features with their *m*/*z* and charge were compared to tryptic milk peptides. The tryptic hydrolysis was performed in silico tolerating two missed cleavages. In addition to the tryptic hydrolysis, a cleavage after the following amino acid was performed in order to consider further enzymatic processes in milk: A, V, L, I, P, M, F, W, G, Q, and T. Acetylation, carbamidomethylation, carbxoyethylation, carboxymethylation, hexosylation, hydroxylation, lactosylation, methylation, oxidation, and phosphorylation were considered as modifications. The protein sequences of the different milk proteins (α-lactalbumin, β-lactoglobulin, BSA, α-s1-casein, α-s2-casein, β-casein, and κ-casein) were obtained as fasta-files from uniprot.org (accessed 13 March 2024). The comparison between the theoretical mass and the feature was set to 0.002%.

## 3. Results

### 3.1. In-Gel Hydrolysis

**Figure 2:**
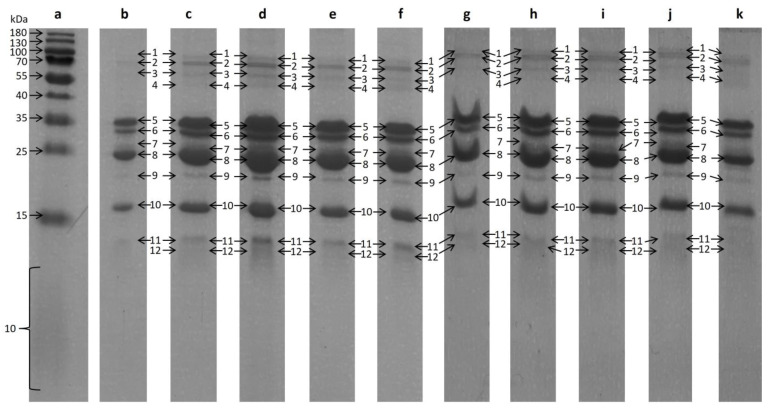
SDS-PAGE patterns of bovine milk, depending on the storing duration; each subsequent lane belongs to a different day of sampling. Marker proteins are shown in lane a. Therefore, lane b–k are assigned day 1 to day 10. The bands in each lane are numbered from 1–12.

Over a period of ten days, a sample of the raw milk was taken each day and analyzed. The results of the SDS-PAGE analysis of the bovine raw milk are shown in Figure 2. One vertical lane represents one sample day. Seven bands (Nos. 2, 5, 6, 8–11) were clearly visible on every lane.

These were cut out of the gel and enzymatically hydrolyzed separately. The enzymatic hydrolysis of the cut outs was conducted with trypsin and analyzed with LC-ESI-IMS-QToF. The acquired data were further investigated using MASCOT. The proteins identified with MASCOT are summarized in Table 1. Three proteins could be clearly identified. Somehow expected, α-s1-casein could be assigned to band No. 5, α-s2-casein could be assigned to band No. 6, and β-lactoglobulin could be assigned to band No. 10. Both β-casein and κ-casein were identified to be present in band No. 8 due to the inadequate separation of band 7 and 8.

**Figure 3:**
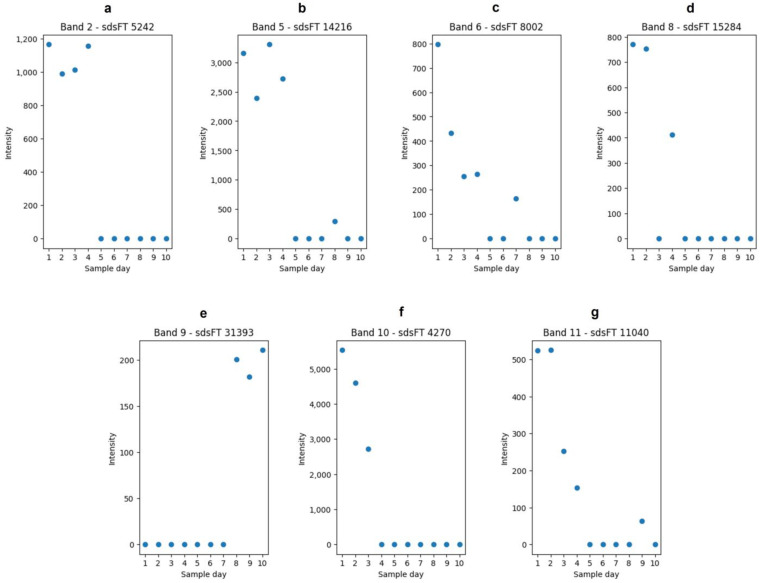
Features selected from the in-gel hydrolysis. Shown are the intensities of the features over the duration of the study. (**a**): Feature “sdsFT 5242” from band No. 2 (*m*/*z*: 242.2844; rt[s]: 654.3949; charge: (1). (**b**): Feature “sdsFT 14216” from band No. 5 (*m/z*: 195.1814; rt[s]: 580.5420; charge: (1). (**c**): Feature “sdsFT 8002” from band No. 6 (*m*/*z*: 518.2820; rt[s]: 803.4882; charge: (2). (**d**): Feature “sdsFT 15284” from band No. 8 (*m*/*z*: 307.0663; rt[s]: 751.2207; charge: 1). (**e**): Feature “sdsFT 31393” from band No. 9 (*m*/*z*: 292.8672; rt[s]: 78.3501; charge: (2). (**f**): Feature “sdsFT 4270” from band No. 6 (*m*/*z*: 430.2453; rt[s]: 468.0191; charge: (1). (**g**): Feature “sdsFT 11040” from band No. 11 (*m*/*z*: 462.8488; rt[s]: 78.9323; charge: (1).

After analyzing the proteins and their band association, the peptides were the focus of further analysis. The peptides were obtained by in-gel hydrolysis of the bands. The aim was to select features that reflect the proteome alteration over the duration of the study. Features can represent any responsible molecule/fragment of a chemical compound. In order to achieve the selection of representative features, features from the mass spectrometric data were extracted and analyzed with chemometric methods. As a chemometric tool, a partial least square regression (PLSR) was used. It was performed for each band separately (Nos 2, 5, 6, 8–11). After performing the PLSR, a VIP score for each feature was calculated. The VIP score is a method to determine the influence of features on the latent variables in a PLS model [38]. As the dataset was rather small, no extra validation was performed. For such a validation, the dataset would be split into two parts, and thereby, datasets for model building are usually decreasing and characteristics in the dataset might get lost. The features with the highest VIP score from each band is shown in Figure 3.

### 3.2. In-Solution Hydrolysis

**Figure 4:**
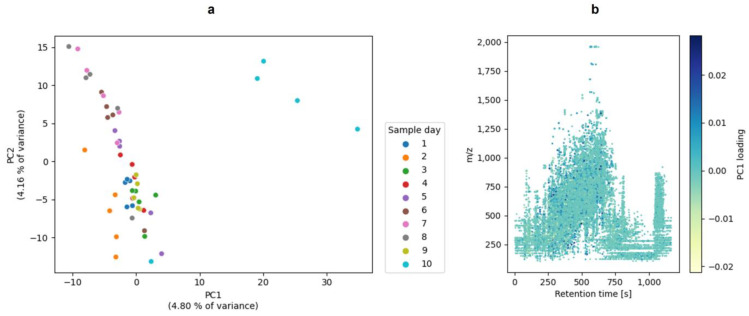
The PCA plot of the in-solution hydrolysis of the milk samples. (**a**): The plot of the scores of principal component 1 (PC1) and principal component 2 (PC2). For the PCA, no further data preprocessing was performed. (**b**): The plot of the loadings of PC1. The plots were generated with matplotlib and seaborn.

In addition to the in-gel hydrolysis, the raw milk samples were enzymatically hydrolyzed in-solution. With the extracted features from the mass spectrometric analysis of the in-solution hydrolysis, a PCA, a PLS-DA, and a PLSR were performed in order to compare these evaluation approaches and, more importantly, select features that reflect the alteration of the proteome. A PCA was applied to obtain an initial overview of the data and as an exploratory data analysis (EDA). Figure 4a visualizes the PCA as a scatter plot showing the scores of principal components 1 (PC1) and 2 (PC2). Figure 4b shows the loadings for the PC 1 features. A PCA is an unsupervised technique that clusters the data based on maximizing the variance. The PCA of the present study showed a separation of the samples. Four samples of sample day 10 were thereby separated from the other samples.

**Figure 5:**
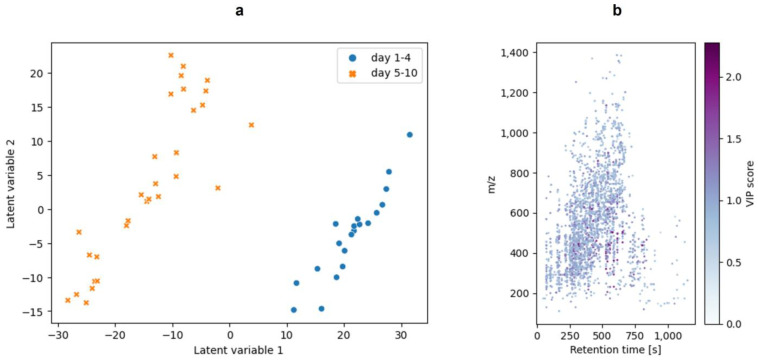
The PLS-DA of the in-solution hydrolysis raw milk samples. The used classes for the PLS-DA were “days 1–4” vs. “days 5–10”. (**a**): The scatter plot of the latent variables of the PLS-DA. The PLS-DA was performed after feature selection based on the VIP scores after a previous PLS-DA. (**b**): The plot of the features with VIP scores after feature selection. These features are also the features that were used for the PLS-DA model in (**a**). The plots were generated with matplotlib and seaborn.

Following the PCA, a PLS-DA was performed. It was used as a classification method for this study. Therefore, the data were split into two categories: ‘days 1–4’ vs. ‘days 5–10’. The split of the data in these two categories was defined based on the use-by date for raw milk, which is 96 h after production [39]. In the present study, the PLS-DA was the preferred classification method, as it well suited for feature selection [38,40,41]. Moreover, the usability of the PLS-DA in a comparable python-based workflow was already demonstrated [31]. In order to select features and reduce the data for the model, the features were filtered by their VIP scores. Only features with a VIP score > 1.5 were used for the PLS-DA. The approach of filtering data by the VIP score before performing a classification was discussed by Christmann et al. (2022), who showed the feasibility for a GC-IMS dataset [42]. Figure 5 shows the visualization of the PLS-DA (Figure 5a) and the VIP scores of the features (Figure 5b). A confusion matrix of the validation is included in the Supplementary Materials (Figure S4). The ten features with the highest VIP scores are listed in Table S1 (Supplementary Materials). Plots of the intensities over the duration of the study are given in Figure S5 (Supplementary Materials). A unique feature that reflects the classification by appearing in only one of the two classes (“days 1–4” vs. “days 5–10”) could not be determined. However, the features showed steadily increasing or decreasing intensities, supporting the evaluation using a regression tool. Regression is suited if a linear correlation between features and target values (sample days) is given, which is indicated by the mentioned features.

**Figure 6:**
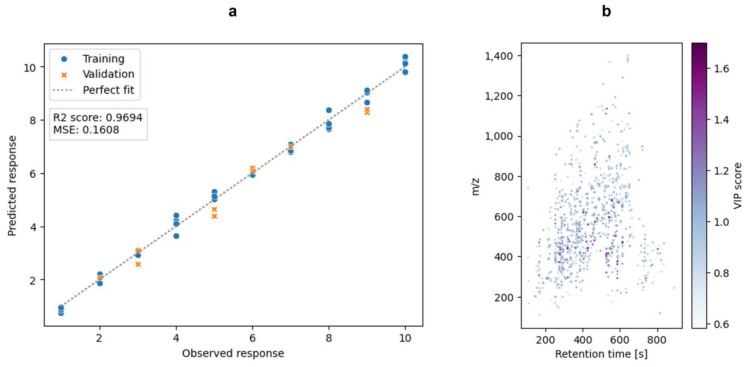
The PLSR of the milk samples (in-solution hydrolysis) over time. Raw milk samples were analyzed over a time of ten days. (**a**): The plot shows the observed response versus the predicted response of the training data (blue dots) and the validation data (orange crosses). (**b**): The plot of the features that were used to build the PLSR model. The plots were generated with matplotlib and seaborn.

As a third chemometric tool, a PLSR was performed to evaluate the data. This is a supervised regression method that is widely used in chemometrics. It can deal with many input variables and noise [43]. For performing the PLSR, the dataset was filtered based on the F-values of the features. A visualization of the PLSR is shown in Figure 6. It shows the performance of the prediction as well as the validation with a mean squared error (MSE) of 0.1608 and a R^2^ of 0.9694.

**Figure 7:**
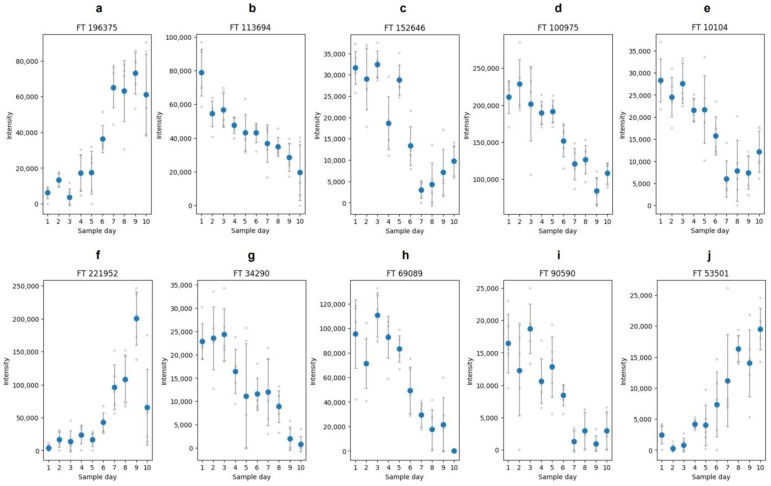
Intensities of selected features over the duration of the study. Shown are the ten features with the highest VIP score from the PLSR of the in-solution samples. The shown features are (**a**): FT 196375; (**b**): FT 113694; (**c**): FT 152646; (**d**): FT 100975; (**e**): FT 10104; (**f**): FT 221952; (**g**): FT 34290; (**h**): FT 69089; (**i**): FT 90590; (**j**): FT 53501. The plots were generated with matplotlib.

The features with the most intense influence on the regression were selected by the calculation of the VIP score. In Table 2, the ten features with the highest VIP scores in the PLSR are listed. Figure 7 shows these Top 10 features and their change over the duration of the study. In general, 50 features were selected, which are all listed in the Supplementary Materials (Table S1). All Top 50 features were further investigated on two main aspects. First, the features were analyzed to determine whether the features were also detected in the mass spectrometric analysis of the in-gel hydrolysis samples. The results of the in-gel hydrolysis were shown above. If a feature is also found in the samples of the in-gel hydrolysis, this can provide an indication about the protein origin due to the band assignment of the in-gel hydrolysis samples. Second, the features were analyzed regarding their peptide sequence and potential modifications of the peptide.

**Figure 8:**
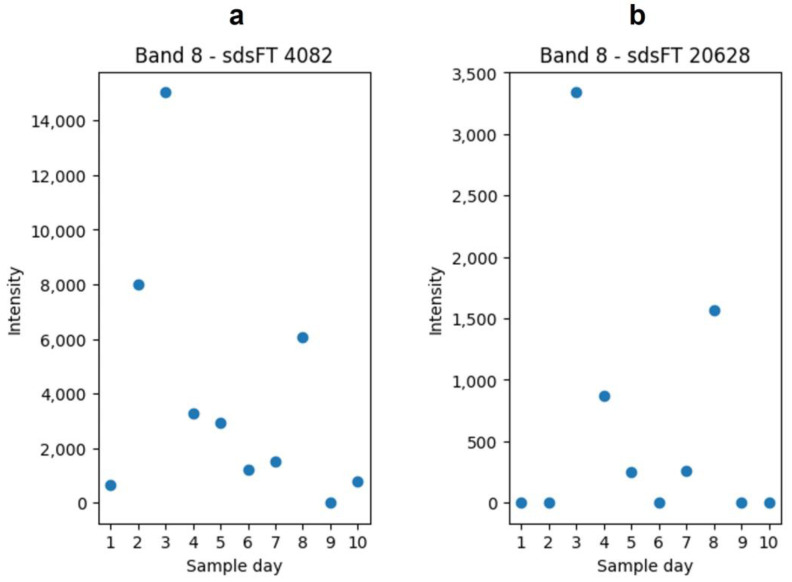
The plot of the intensities of feature sdsFT 4082 (**a**) and sdsFT 20,628 (**b**). Both features were detected in the in-solution hydrolysis and in-gel hydrolysis. The plotted intensities are from the in-gel hydrolysis in the band No. 8. The plots were generated with matplotlib.

The results of the first part, the comparison with the features of the in-gel hydrolysis, are listed in Table 2 as column “SDS-PAGE”. In Table 2, there are two features that are particularly striking. FT 100,975 and FT 113,694 had a similar retention time in the UPLC-MS analysis and are both found in the in-solution hydrolysis samples as well as in the in-gel hydrolysis samples. The difference between the neutral masses of these two features can be determined as 46.9994 Da. The features were detected in in the SDS-PAGE as sdsFT 4082 and sdsFT 20628, both appearing in band No. 8 (Figure 2), which was identified as β- and κ-casein (Table 1). The intensities of these features in the in-gel hydrolysis experiment are shown in Figure 8. The decreasing intensity over the course of the milk storage can also be recognized in the in-gel hydrolysis samples but with a less clear picture and increased scattering. Based on these similarities, it can be assumed that both features have a similar source of origin.

The molecular identification of the features, which were selected after the PLSR, was based on an approach presented in a recent study [31]. Different factors in raw bovine milk have to be considered for the identification process. These factors have an influence on the formation or presence of certain peptides and should therefore be considered when determining the peptides to be considered for identification. One of these factors is that naturally, raw bovine milk contains proteases. These proteases hydrolyse the proteins, leading to endogenous peptides. The most relevant are proteases such as plasmin, cathepsin D, elastase, and cathepsin B [32]. Moreover, further modifications of peptides like posttranslational modifications (PTM) should be taken into account as well because they can modify the peptides and proteins during the study and might act as potential markers [25]. Acetylation, carbamidomethylation, carboxyethylation, carboxymethylation, hexosylation, hydroxylation, lactosylation, methylation, oxidation, and phosphorylation were considered as PTM in the present study. From the selected features (Supplementary Materials, Table S1), three features could be identified and are listed in Table 3.

## 4. Discussion

The present study demonstrated the analysis of the proteoforms of raw milk using different sample preparation as well as different data analysis methods. In-gel hydrolysis and in-solution hydrolysis were compared. Both are common techniques in proteomics [44]. The integration of gel electrophoresis for analyzing and, above all, separating (milk) proteins is a widely used tool [45,46,47]. Jovanovic et al. analyzed soluble proteins in reconstituted milk with an SDS-PAGE analysis [45]. They showed that α-s1-casein and α-s2-casein as well as β-casein and κ-casein are difficult to separate from each other in the gel, which is a phenomenon that was also observed in the present study [45]. Holzmüller et al. described the quantification of milk fat globule membrane proteins in buttermilk and butter serum [46]. They presented a modified version of an SDS-PAGE in order to avoid the affinity problematic of the dyes [46]. In the present study as well as in the study described by Holzmüller et al., caseins and β-lactoglobulin were detected in the SDS-PAGE. In contrast to Holzmüller et al., a conventional staining method with Coomassie Brilliant Blue was used in the present study, as the aim was identification rather than quantitatification, and the unstable affinity of the staining was therefore accepted.

The advantage of pre-separating proteins is the simplification of the interpretation of the mass spectrometric data, as there are less peptides present, and for these peptides, potentially, a common protein source can be assigned. However, the 1D separation of the proteins was not sufficient to adequately separate all milk proteins from each other, and thereby, an assignment of the band to one protein was not always possible. In general, there is more potential for quantitative losses in the process of in-gel hydrolysis compared to the in-solution hydrolysis [48]. Such losses can influence the quality of the identification of proteins extracted from the gel. This could explain why not all milk proteins were detected evenly in all sample days in the presented study. Over the duration of sampling, natural degradation processes of the proteins in the milk take place, and the concentration of proteins decreases over time. As natural degradation processes in milk different proteases, e.g., plasmin or cathepsin D, are described. These milk proteases occur naturally in the milk and degrade specific proteins [49,50]. Such processes could cause that to the end of the study less proteins are identified with MASCOT. In general, the in-gel hydrolysis is significantly more time- and material-consuming as the in-solution hydrolysis of milk.

In general, the peptidome of milk has been analyzed before with the aim of monitoring the storage and processing of milk. In previous studies described by Class et al. and Kuhnen et al., methodologies were established that were also used in the present study [5,31]. Moreover, further researchers analyzed the peptidome to answer questions regarding different processing techniques and storage experiments [2,23]. The processing of milk was analyzed, for example, by Wölk et al., who compared raw milk with ultra-high-temperature (UHT) milk and infant formula [32]. In another study, Wölk et al. compared regular and hay milk and analyzed the influence of processing [51]. Furthermore, the influence of heat and processing was investigated by Ebner et al. with a study comparing high-temperature short-time milk, extended-shelf-life milk, and ultra-high-temperature-milk [25]. Another approach in the context of a peptidomic-based analysis of milk spoilage is to focus on the activity of specific bacteria that cause spoilage. One example for this is the study described by Stuknyté et al., who focused on peptides that indicate the activity of *Pseudomonas fluorenscens* PS19 in milk [52]. The variability of the proteome and its ability to reflect external influences is not only used in the context of milk but can be also applied to other studies for investigating peptide markers regarding the spoilage or shelf-life of various matrices. For instance, Chen et al. investigated the shelf-life of pacific oysters and proposed four endogenous peptides as potential markers [4]. In another example by Mentana et al., the peptide profile was also used to analyze the product quality of Italian soft cream cheese [53]. Different storage conditions were investigated, and the peptide profile was analyzed using nano-LC-MS/MS in combination with a novel bioinformatics approach [53]. Mentana et al. observed an increase in peptides derived from α-s_1_-casein [53]. Wei et al. used a biomarker approach in the context of meat freshness [22]. In that study, they analyzed pork meat in order to determine potential biomarkers for its spoilage [22]. Further examples in the context of pork spoilage are presented by Zou et al. and Zhao et al. [54,55]. Furthermore, a biomarker-based analysis of food spoilage can be also performed with a focus of peptide releases as exotoxins by pathogens [56].

In the present study, features, which were extracted from the in-gel hydrolysis experiment, can be compared with features from the in-solution hydrolysis. From both datasets, characteristic features were extracted with PLSR and VIP scores. The extracted features from the in-gel hydrolysis showed a less clear regression than the features extracted from the in-solution hydrolysis. The features that were extracted from the in-solution hydrolysis are potential marker peptides for the alteration of the proteome. Their potential as marker peptides is based on their correlation between the sample day and the intensity of the features, which was observed. The features that were extracted from the in-solution hydrolysis were compared with the features from the in-gel hydrolysis. Thereby, for three features from the in-solution hydrolysis, similar features in the in-gel hydrolysis samples were found. Table 2 shows these features. As the features are similar, a common peptide source can be assumed and thereby a common protein source. In the in-gel hydrolysis, the three features were found in band No. 8, which was technically identified as β- and κ-casein. Therefore, these three features were assumed to be from one of these two proteins. This demonstrates how the in-gel hydrolysis and the in-solution hydrolysis can be used in combination, and the results can be compared.

The approach for the identification was transferred from a different study, where milk peptides from UHT milk were identified [5]. It was adapted to the present study by adjusting the database of the considered peptides, which were calculated after a theoretic tryptic hydrolysis of milk proteins. For a theoretic digest in the present study, more cleavage sides (from the milk proteases) were considered in the process of calculating the peptides. Furthermore, possible modifications were adjusted based on the literature. The identification was performed with the selected features, being of a chemical nature of peptides. Three features selected from the PLSR were identified (Table 3). Two of them (FT 62883 and FT 298105) were identified as the same phosphorylated but differentially charged peptide FSDIPNPIGSENSEK from the milk protein α-s1-casein. Phosphorylation is one of the most common PTM proteome-wide [57]. The phosphorylation of peptides can occur due to enzymatic processes in the milk and was analyzed by Baum et al. [58]. In addition to the two phosphorylated features, feature FT 128885 was identified as the peptide PLW from α-s1-casein. The small number of identified features in the present study makes it clear that the assignment of peptides/features is not easy.

The proteome and, with it, the peptidome of raw milk are influenced by diverse reactions and modifications during storage. Part of these reactions are endogenous proteases like plasmin or cathepsin D, which are naturally present in the milk [23,50,59]. For the selected features, potential matches based on the mass of the feature were found. However, for only a limited number of features, the identification could be confirmed based on the fragmentation of the peptide (b- and y-fragments). A reason for the difficulties in the identification can be that the fragments were acquired with MS^E^, which is a type of data-independent acquisition [60]. Fragments of different precursors are in a single high-energy spectrum and can interfere with the identification process.

In the present study, three features were identified as peptides from a-s1-casein (Table 3), and three other features could be determined as originating from β- or κ-casein (Table 2). These casein-derived features go along with the reported role of caseins as markers in the change of the milk proteome. The caseins and peptides derived from caseins play an important role especially for potential markers of changes in the proteome, as different studies show [23,25,61,62]. One example for this is the study described by Mansor et al. [61]. They found mainly β- and α-s1-casein-derived peptides as potential biomarkers for bovine mastitis [61]. A different example is described by Ebner et al., who also identified potential biomarkers for the heat treatment from α- and β-caseins [25]. In the study of Van Vlierberghe et al., which was also about the selection of processing markers for milk, seven of the eight identified peptide markers could be assigned to the casein fraction [62]. Dalabasmaz et al. also analyzed the peptide profile of milk for identifying storage markers, mainly considering peptides produced by milk proteases or bacterial proteases, which are endogenously present in the milk [23]. In that study, it was striking that the main markers originate from only β-casein [23]. In general, other studies indicate that whey proteins are less relevant as peptide precursors [32,63], e.g., β-lactoglobulin is, according to Wölk et al., less susceptible to degradation by proteases.

## 5. Conclusions

The present study showed different ways to select structural features/peptides that represent a change in the proteome during storage at the hand of the example raw milk. It was used for an exemplary analytical method development, with the model being transferable to different matrices in future studies (e.g., the alteration of plant-based proteins during processing). Due to the relatively fast spoilage and the already existing studies and knowledge on its proteins, raw milk offers an excellent basis for method development, as it was shown in this study. However, a fish quality analysis can also live from such a proteomic, bioinformatic approach. Also, peptides might be markers for spoilage and changes during storage or ripening.

The present study compared the in-solution and in-gel hydrolysis of the samples as different ways for sample preparation. More information was obtained from the in-solution hydrolysis than from the in-gel hydrolysis. In general, the separation of the proteins prior to enzymatic hydrolysis (in-gel hydrolysis) represents an enormous additional effort compared to a direct hydrolysis of the sample in the solution.

The different chemometric tools were used to select different types of features. A PCA, a PLS-DA, and a PLSR were used. The PLSR was chosen to select features. Fifty features were selected using the PLSR in combination with VIP scores. In the present study, peptides were mainly focused for being potential markers for the shelf-life of raw milk. Three of these features were identified as promising marker peptides. The usability of the features as routine markers should be analyzed in further studies. Of course, a non-targeted mass spectrometry approach as a routine analysis method seems to be inappropriate, but identifying unique peptides (patterns) or even developing fast analysis methods might enable a more robust targeted analysis. When performing mass spectrometry as a routine analysis, artificial intelligence might be helpful to monitor larger or smaller sets of peptides for use in (milk) quality control.

## Figures and Tables

**Table 1:** The results of the band identification with MASCOT.

Sample Day	Band Number	MASCOT Identification	Scores
1	-	-	-
2	5	α-s1-Casein	48
	8	κ-Casein	75
	8	β-Casein	56
3	5	α-s1-Casein	85
	6	α-s2-Casein	62
	8	κ-Casein	111
	8	β-Casein	62
	10	β-Lactoglobulin	167
4	5	α-s1-Casein	67
	8	κ-Casein	76
	10	β-Lactoglobulin	73
5	5	α-s1-Casein	94
	8	κ-Casein	63
	8	β-Casein	54
	10	β-Lactoglobulin	60
6	-	-	-
7	5	α-s1-Casein	70
	8	κ-Casein	66
	8	β-Casein	50
	10	β-Lactoglobulin	40
8	5	α-s1-Casein	86
	8	κ-Casein	60
	8	β-Casein	68
	10	β-Lactoglobulin	79
9	10	β-Lactoglobulin	193
10	10	β-Lactoglobulin	42

**Table 2:** Top ten features selected for the PLSR of the in-solution hydrolysis samples. The features are selected based on their VIP score. The indication “Increase/Decrease” refers to the development of the intensities of the features over the course of the study. In the column, “SDS-PAGE” is listed if a feature was found in the in-gel hydrolysis samples as well.

Feature Name	*m*/*z*	rt [s]	Charge	VIP Score	Increase (I)/ Decrease (D)	SDS-PAGE
FT 196375	322.9788	230.3010	1	1.6993	I	-
FT 113694	399.6896	587.1291	2	1.6491	D	Band 8—sdsFT20628
FT 152646	472.1981	614.6866	2	1.6319	D	-
FT 100975	376.1899	587.4633	2	1.6277	D	Band 8—sdsFT4082
FT 10104	415.1563	614.5459	2	1.6210	D	-
FT 221952	620.3291	403.8281	2	1.6129	I	-
FT 34290	442.1821	320.1580	2	1.5732	D	Band 8—sdsFT1284
FT 69089	594.6089	543.7169	3	1.5687	D	-
FT 90590	502.1869	577.8206	1	1.5678	D	-
FT 53501	411.0683	278.2279	1	1.5637	I	-

**Table 3:** Identified features from the in-solution hydrolysis. The features are listed with their feature name, *m*/*z*, retention time, charge, and if the intensity of the feature increased or decreased during the study. The assigned peptide is listed with modification (if applicable), amino acid (AA) sequence, and protein source.

Feature Name	*m*/*z*	rt [s]	Charge	Increase (I)/ Decrease (D)	Modification	AA Sequence	Protein	Position
FT 128885	415.2385	538.6355	1	D	-	PLW	α-s1-Casein	212–214
FT 62883	857.3572	465.8155	2	D	phosphory-lation	FSDIPNPIGSENSEK	α-s1-Casein	194–208
FT 298105	571.9066	465.9806	3	D	phosphory-lation	FSDIPNPIGSENSEK	α-s1-Casein	194–208

## Data Availability

The original contributions presented in the study are included in the article and Supplementary Materials; further inquiries can be directed to the corresponding authors.

## Supplementary Materials

The following supporting information can be downloaded at https://www.mdpi.com/article/10.3390/foods13203315/s1, Figure S1: The evaluation of the optimal number of PLS components for the PLSR of the in-solution hydrolysis dataset. Top graph shows the calculated MSE depending on the number of PLS components. The graph on the bottom shows the R2 depending on the number of PLS components. The plot was generated with matplotlib; Figure S2: SDS-PAGE-Gel visualizing the protein mass marker, day 1–day 5 from the left to the right; Figure S3: SDS-PAGE-Gel visualizing the protein mass marker, day 6–day 10 from the left to the right; Figure S4: The validation matrix of the PLS-DA of the in-solution-digest raw milk samples. The used classes for the PLS-Da were ‘day 1–4′ (label 0) vs. ‘day 5–10′ (label 1). The PLS-DA was performed after feature selection based on the VIP scores after a previous PLS-DA. The plot was generated with scikit-learn (ConfusionMatrixDisplay); Figure S5: Intensities of selected features over the duration of the study. Shown are the ten features with the highest VIP score from the PLS-DA (“day 1–4” vs. “day 5–10”) of the in-solution hydrolysis samples. The plots were generated with matplotlib; Table S1: Top ten features selected for the PLS-DA (“day 1–4” vs. “day 5–10”) of the in-solution hydrolysis samples. The features are selected based on their VIP score; Table S2: Top 50 features selected for the PLSR of the milk samples. The features listed have the VIP scores.

## References

[1] Solazzo C., Wadsley M., Dyer J.M., Clerens S., Collins M.J., Plowman J. (2013). Characterisation of novel α-keratin peptide markers for species identification in keratinous tissues using mass spectrometry. Rapid Commun. Mass Spectrom..

[2] von Oesen T., Treblin M., Clawin-Rädecker I., Martin D., Maul R., Hoffmann W., Schrader K., Wegner B., Bode K., Zink R. (2023). Identification of Marker Peptides for the Whey Protein Quantification in Edam-Type Cheese. Foods.

[3] Chassaigne H., Nørgaard J.V., Van Hengel A.J. (2007). Proteomics-based approach to detect and identify major allergens in processed peanuts by capillary LC-Q-TOF (MS/MS). J. Agric. Food Chem..

[4] Chen L., Zhang H., Zhang X., Yu F., Zhang F., Xue C., Xue Y., Tang Q., Li Z. (2020). Identification of potential peptide markers for the shelf-life of Pacific oysters (*Crassostrea gigas*) during anhydrous preservation via mass spectrometry-based peptidomics. LWT Food Sci. Technol..

[5] Class L.-C., Kuhnen G., Hanisch K.L., Badekow S., Rohn S., Kuballa J. (2024). The Shelf Life of Milk—A Novel Concept for the Identification of Marker Peptides Using Multivariate Analysis. Foods.

[6] Augustin M.A., Riley M., Stockmann R., Bennett L., Kahl A., Lockett T., Osmond M., Sanguansri P., Stonehouse W., Zajac I. (2016). Role of food processing in food and nutrition security. Trends Food Sci. Technol..

[7] Jedermann R., Nicometo M., Uysal I., Lang W. (2014). Reducing food losses by intelligent food logistics. Philos. Trans. R. Soc. A Math. Phys. Eng. Sci..

[8] Corradini M.G. (2018). Shelf Life of Food Products: From Open Labeling to Real-Time Measurements. Annu. Rev. Food Sci. Technol..

[9] Class L.-C., Kuhnen G., Rohn S., Kuballa J. (2021). Diving Deep into the Data: A Review of Deep Learning Approaches and Potential Applications in Foodomics. Foods.

[10] Stenmarck Å., Jensen C., Quested T., Moates G. (2016). FUSIONS—Estimates of European Food Waste Levels.

[11] Toma L., Costa Font M., Thompson B. (2020). Impact of consumers’ understanding of date labelling on food waste behaviour. Oper. Res..

[12] Patra D., Feng S., Howard J.W. (2022). Confusion of food-date label with food safety—implications for food waste. Curr. Opin. Food Sci..

[13] Madilo F.K., Owusu-Kwarteng J., Parry-Hanson Kunadu A., Tano-Debrah K. (2020). Self-reported use and understanding of food label information among tertiary education students in Ghana. Food Control.

[14] Priefer C., Jörissen J., Bräutigam K.R. (2016). Food waste prevention in Europe—A cause-driven approach to identify the most relevant leverage points for action. Resour. Conserv. Recycl..

[15] Kavanaugh M., Quinlan J.J. (2020). Consumer knowledge and behaviors regarding food date labels and food waste. Food Control.

[16] Buttlar B., Löwenstein L., Geske M.S., Ahlmer H., Walther E. (2021). Love food, hate waste? Ambivalence towards food Fosters people’s willingness to waste food. Sustainability.

[17] Martin N.H., Ranieri M.L., Murphy S.C., Ralyea R.D., Wiedmann M., Boor K.J. (2011). Results from raw milk microbiological tests do not predict the shelf-life performance of commercially pasteurized fluid milk. J. Dairy Sci..

[18] Condurso C., Cincotta F., Tripodi G., Merlino M., Giarratana F., Verzera A. (2020). A new approach for the shelf-life definition of minimally processed carrots. Postharvest Biol. Technol..

[19] Dalgaard P. (1995). Modelling of microbial activity and prediction of shelf life for packed fresh fish. Int. J. Food Microbiol..

[20] Machado S.G., Baglinière F., Marchand S., Van Coillie E., Vanetti M.C.D., De Block J., Heyndrickx M. (2017). The biodiversity of the microbiota producing heat-resistant enzymes responsible for spoilage in processed bovine milk and dairy products. Front. Microbiol..

[21] Verhegghe M., De Block J., Heyndrickx M., Van Coillie E., Van Poucke C., Duquenne B. (2021). Application of LC-HRMS identified marker peptides in an LC-MS/MS method for detection and quantification of heat-resistant proteolytic activity in raw milk. Int. J. Dairy Technol..

[22] Wei Z., Dai C., Bassey A.P., Tang C., Han Y., Wang C., Zhou G. (2022). Identification of Potential Peptide Marker(s) for Evaluating Pork Meat Freshness via Mass Spectrometry-Based Peptidomics during Storage under Different Temperatures. Foods.

[23] Dalabasmaz S., Dittrich D., Kellner I., Drewello T., Pischetsrieder M. (2019). Identification of peptides reflecting the storage of UHT milk by MALDI-TOF-MS peptide profiling. J. Proteomics.

[24] Liu H., Grosvenor A.J., Li X., Wang X.l., Ma Y., Clerens S., Dyer J.M., Day L. (2019). Changes in Milk Protein Interactions and Associated Molecular Modification Resulting from Thermal Treatments and Storage. J. Food Sci..

[25] Ebner J., Baum F., Pischetsrieder M. (2016). Identification of sixteen peptides reflecting heat and/or storage induced processes by profiling of commercial milk samples. J. Proteomics.

[26] Pischetsrieder M., Baeuerlein R. (2009). Proteome research in food science. Chem. Soc. Rev..

[27] Gross J.H. (2013). Massenspektrometrie—Ein Lehrbuch.

[28] Shevchenko A., Tomas H., Havliš J., Olsen J.V., Mann M. (2007). In-gel digestion for mass spectrometric characterization of proteins and proteomes. Nat. Protoc..

[29] Pedreschi R., Maarten H., Lilley K.S., Bart N. (2010). Proteomics for the food industry: Opportunities and challenges. Crit. Rev. Food Sci. Nutr..

[30] Matissek R., Fischer M. (2021). Lebensmittelanalytik.

[31] Kuhnen G., Class L.C., Badekow S., Lara K., Sascha H., Jürgen R. (2024). Python workflow for the selection and identification of marker peptides—proof-of-Principle study with heated milk. Anal. Bioanal. Chem..

[32] Wölk M., Milkovska-Stamenova S., Hoffmann R. (2020). Comprehensive profiling of the native and modified peptidomes of raw bovine milk and processed milk products. Foods.

[33] Giansanti P., Tsiatsiani L., Low T.Y., Heck A.J.R. (2016). Six alternative proteases for mass spectrometry-based proteomics beyond trypsin. Nat. Protoc..

[34] Shevchenko A., Wilm M., Vorm O., Mann M. (1996). Mass spectrometric sequencing of proteins from silver-stained polyacrylamide gels. Anal. Chem..

[35] Chambers M.C., MacLean B., Burke R., Amodei D., Ruderman D.L., Neumann S., Gatto L., Fischer B., Pratt B., Egertson J. (2012). A cross-platform toolkit for mass spectrometry and proteomics. Nat. Biotechnol..

[36] Van Rossum G., Drake F.L. (2014). The Python Language Reference.

[37] Scikit-Learn Developers sklearn.feature_selection.SelectKBest. https://scikit-learn.org/stable/modules/generated/sklearn.feature_selection.SelectKBest.html.

[38] Farrés M., Platikanov S., Tsakovski S., Tauler R. (2015). Comparison of the variable importance in projection (VIP) and of the selectivity ratio (SR) methods for variable selection and interpretation. J. Chemom..

[39] (2007). Bundesamt für Verbraucherschutz und Lebensmittelsicherheit (BVL) Verordnung über Anforderungen an die Hygiene beim Herstellen, Behandeln und Inverkehrbringen von Bestimmten Lebensmitteln Tierischen Ursprungs (Tierische Lebensmittel-Hygieneverordnung—Tier-LMHV).

[40] Mehmood T., Sæbø S., Liland K.H. (2020). Comparison of variable selection methods in partial least squares regression. J. Chemom..

[41] Mehmood T., Ahmed B. (2016). The diversity in the applications of partial least squares: An overview. J. Chemom..

[42] Christmann J., Rohn S., Weller P. (2022). Finding features—Variable extraction strategies for dimensionality reduction and marker compounds identification in GC-IMS data. Food Res. Int..

[43] Wold S., Sjöström M., Eriksson L. (2001). PLS-regression: A basic tool of chemometrics. Chemom. Intell. Lab. Syst..

[44] Ortea I., O’Connor G., Maquet A. (2016). Review on proteomics for food authentication. J. Proteomics.

[45] Jovanovic S., Barac M., Macej O., Vucic T., Lacnjevac C. (2007). SDS-PAGE analysis of soluble proteins in reconstituted milk exposed to different heat treatments. Sensors.

[46] Holzmüller W., Kulozik U. (2016). Quantification of MFGM proteins in buttermilk and butter serum by means of a stain free SDS-PAGE method. J. Food Compos. Anal..

[47] Kausar R., Hameed A., Qureshi Z., Muhammd G. (2016). Comparative Protein Profiling of Milk of Nili-Ravi Buffaloes, Sahiwal and Cross Bred Cows by SDS-PAGE. Pak. Vet. J..

[48] Havliš J., Thomas H., Šebela M., Shevchenko A. (2003). Fast-Response Proteomics by Accelerated In-Gel. Anal. Chem..

[49] Andrews A.T. (1982). Proteinases in normal bovine milk and their action on caseins. J. Diary Res..

[50] Nielsen S.S. (2002). Plasmin system and microbial proteases in milk: Characteristics, roles, and relationship. J. Agric. Food Chem..

[51] Wölk M., Milkovska-Stamenova S., Schröter T., Hoffmann R. (2021). Influence of seasonal variation and processing on protein glycation and oxidation in regular and hay milk. Food Chem..

[52] Stuknytė M., Decimo M., Colzani M., Silvetti T., Brasca M., Cattaneo S., Aldini G., Noni I. (2016). De Extracellular thermostable proteolytic activity of the milk spoilage bacterium Pseudomonas fluorescens PS19 on bovine caseins. J. Diary Sci..

[53] Mentana A., Natale A., Palermo C., Nardiello D., Conte A., Del Nobile M.A., Quinto M., Centonze D. (2016). Mass spectrometry hyphenated techniques for the analysis of volatiles and peptides in soft cheese: Useful tools for the shelf life optimization. Electrophoresis.

[54] Zhao F., Wei Z., Bai Y., Li C., Zhou G., Kristiansen K., Wang C. (2022). Proteomics and Metabolomics Profiling of Pork Exudate Reveals Meat Spoilage during Storage. Metabolites.

[55] Zou X., He J., Zhao D., Zhang M., Xie Y., Dai C., Wang C., Li C. (2020). Structural Changes and Evolution of Peptides During Chill Storage of Pork. Front. Nutr..

[56] Böhme K., Fernández-No I.C., Calo-Mata P., Barros-Velázquez J. (2017). Proteomics of Food Spoilage Pathogens.

[57] Khoury G.A., Baliban R.C., Floudas C.A. (2011). Proteome-wide post-translational modification statistics: Frequency analysis and curation of the swiss-prot database. Sci. Rep..

[58] Baum F., Ebner J., Pischetsrieder M. (2013). Identification of Multiphosphorylated Peptides in Milk. J. Agric. Food Chem..

[59] Hurley M.J., Larsen L.B., Kelly A.L., Mcsweeney P.L.H. (2001). The milk acid proteinase cathepsin D: A review. Int. Dairy J..

[60] Plumb R.S., Johnson K.A., Rainville P., Smith B.W., Wilson I.D., Castro-Pere J.M., Nicholson J.K. (2006). UPLC/MSE; a new approach for generating molecular fragment information for biomarker structure elucidation. Rapid Commun. Mass Spectrom..

[61] Mansor R., Mullen W., Albalat A., Zerefos P., Mischak H., Barrett D.C., Biggs A., Eckersall P.D. (2013). A peptidomic approach to biomarker discovery for bovine mastitis. J. Proteomics.

[62] Van Vlierberghe K., Gavage M., Dieu M., Renard P., Arnould T., Gillard N., Coudijzer K., De Loose M., Gevaert K., Van Poucke C. (2022). Selecting Processing Robust Markers Using High-Resolution Mass Spectrometry for the Detection of Milk in Food Products. J. AOAC Int..

[63] Baum F., Fedorova M., Ebner J., Hoffmann R., Pischetsrieder M. (2013). Analysis of the endogenous peptide profile of milk: Identification of 248 mainly casein-derived peptides. J. Proteome Res..

