# Supplementary material for: Marker Peptides for Indicating the Spoilage of Milk—Sample Preparation and Chemometric Approaches for Yielding Potential Peptides in a Raw Milk Model
Source: Foods. 2024 Oct 18;13(20):3315. doi: 10.3390/foods13203315 (PMC11507367; doi:10.3390/foods13203315)
Supplement: Supplementary file 1 [file foods-13-03315-s001.zip › foods-3207807-supplementary.pdf]

Article

Marker Peptides for Indicating the Spoilage of Milk—Sample Preparation and Chemometric Approaches for Yielding Potential Peptides in a Raw Milk Model

Lisa-Carina Class [1,2,†], Gesine Kuhnen [1,3,†], Jasmin Schmid [1], Sascha Rohn [3] and Jürgen Kuballa [1,*]

[1] GALAB Laboratories GmbH, Am Schleusengraben 7, 21029 Hamburg, Germany
[2] Hamburg School of Food Science, Institute of Food Chemistry, University of Hamburg, Grindelallee 117, 20146 Hamburg, Germany
[3] Department of Food Chemistry and Analysis, Institute of Food Technology and Food Chemistry, Technische Universität Berlin, Gustav-Meyer-Allee 25, 13355 Berlin, Germany
* Correspondence: juergen.kuballa@galab.de
† These authors contributed equally to this work.

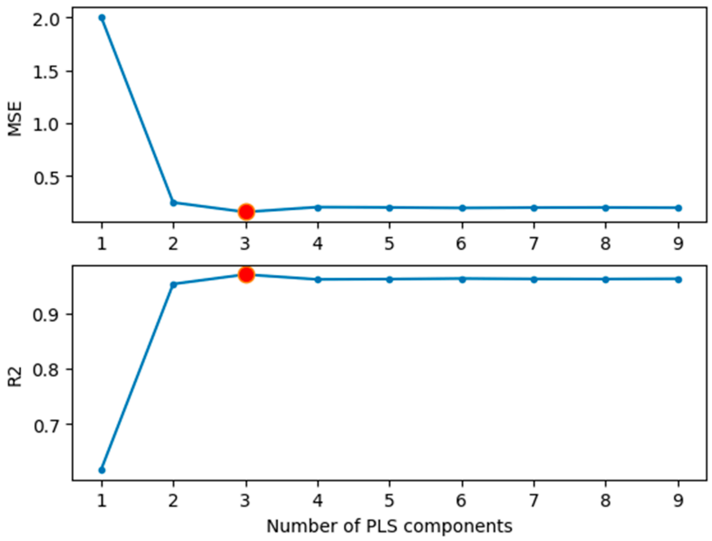

Figure S1*:* Evaluation of the optimal number of PLS components for the PLSR of the in-solution hydrolysis dataset. Top graph shows the calculated MSE depending on the number of PLS components. The graph on the bottom shows the R^2 depending on the number of PLS components. The plot was generated with matplotlib.

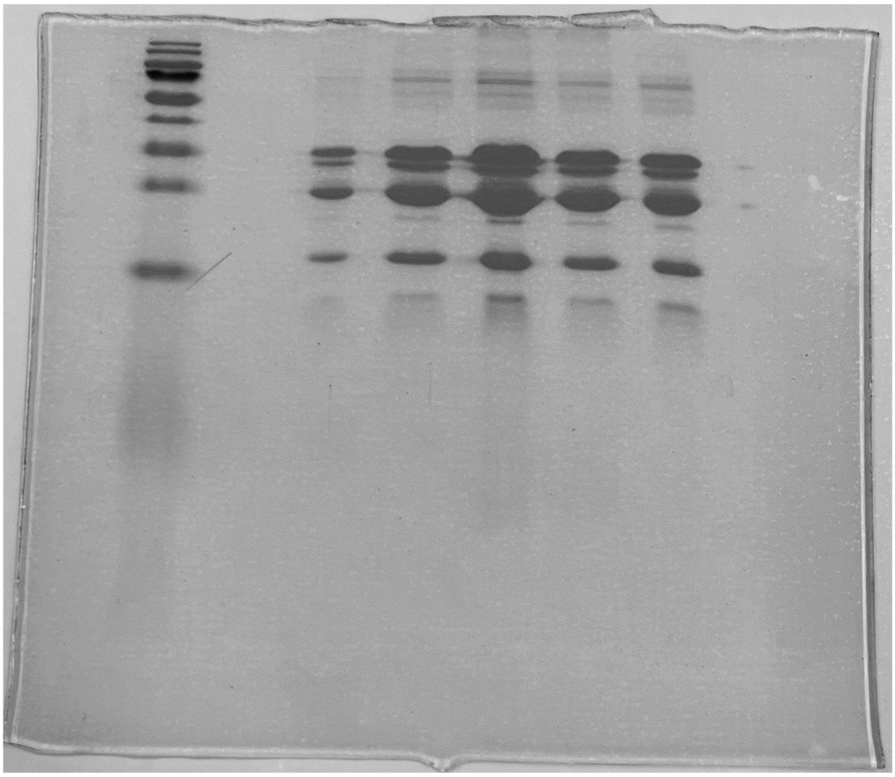

Figure S2*:* SDS-PAGE-gel full scan of the original image which is shown in figure 2 in the paper. The SDS-PAGE-gel is visualizing the protein mass marker, day 1 – day 5 from the left to the right.

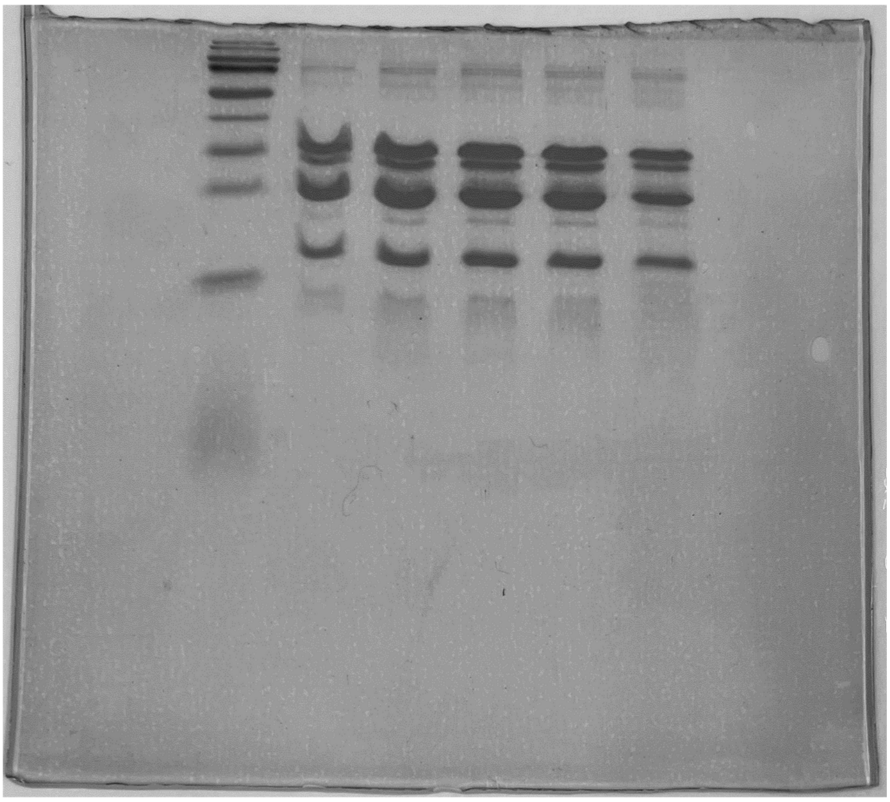

Figure S3*:* SDS-PAGE-gel full scan of the original image which is shown in figure 2 in the paper. The SDS-PAGE-gel is visualizing the protein mass marker, day 6 – day 10 from the left to the right.

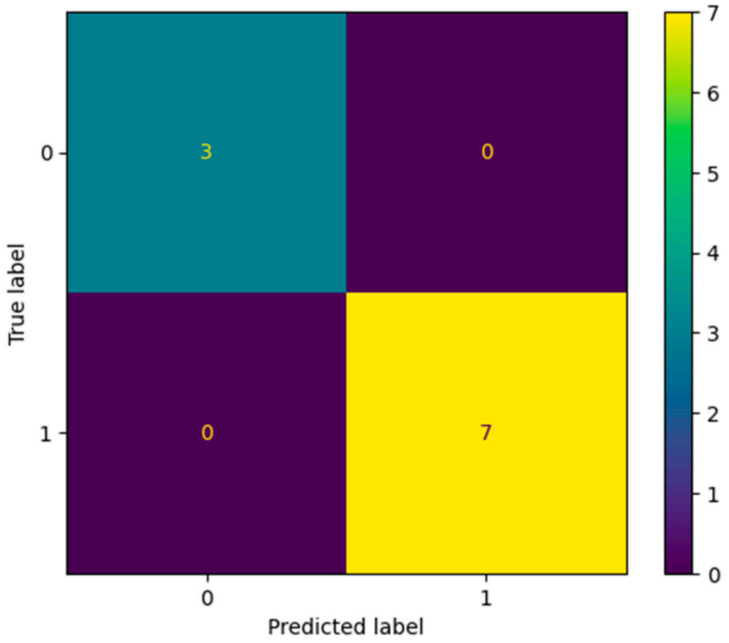

Figure S*4:* Validation matrix of the PLS-DA of the in-solution-digest raw milk samples. The used classes for the PLS-Da were 'day 1-4' (label 0) vs. 'day 5-10' (label 1). The PLS-DA was performed after feature selection based on the VIP scores after a previous PLS-DA. The plot was generated with scikit-learn (*ConfusionMatrixDisplay).*

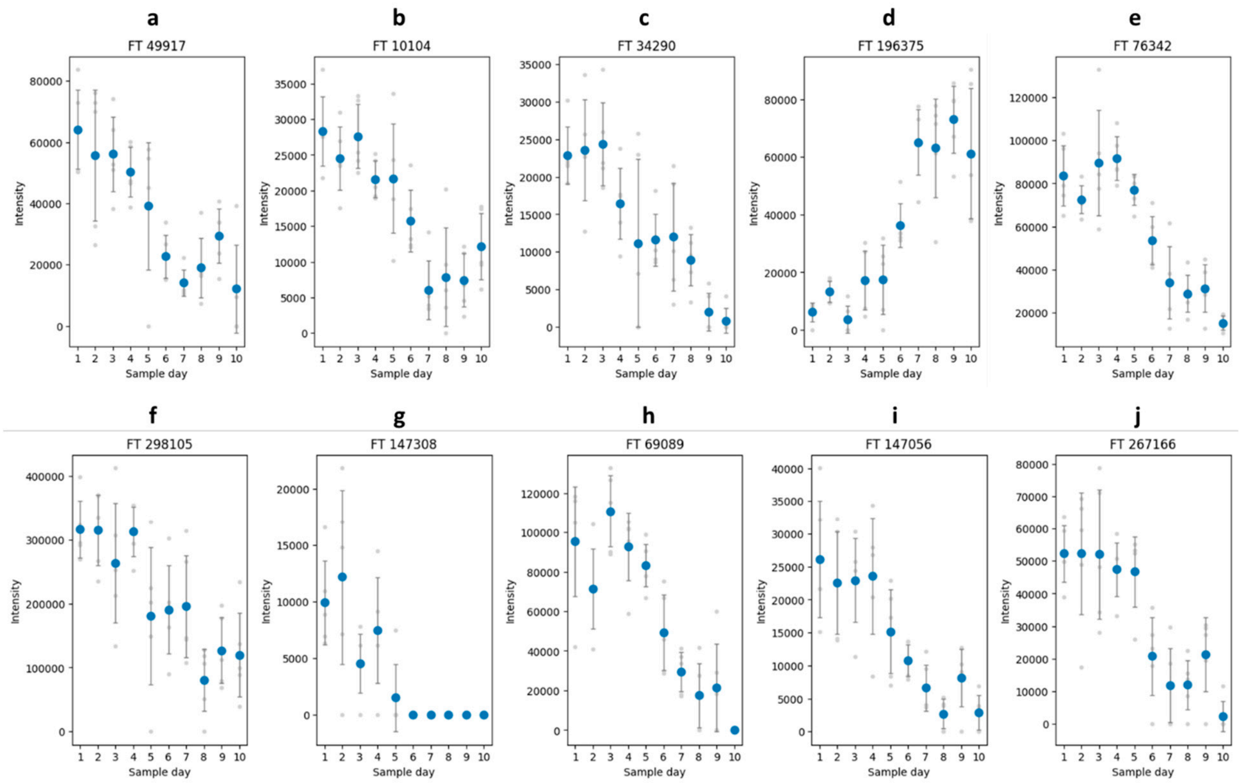

Figure S5*:* Intensities of selected features over the duration of the study. Shown are the ten features with the highest VIP score from the PLS-DA (“day 1-4” vs “day 5-10”) of the in-solution hydrolysis samples. The plots were generated with matplotlib.

Table S1: Top ten features selected for the PLS-DA ("day 1-4" vs "day 5-10") of the in-solution hydrolysis samples. The features are selected based on their VIP score.

Feature name	*m*/*z*	rt [s]	charge	VIP score
FT 49917	379.1337	538.4349	2	2.2772
FT 10104	415.1563	614.5459	2	2.2591
FT 34290	442.1821	320.1580	2	2.1948
FT 196375	322.9788	230.3010	1	2.1829
FT 76342	677.3095	558.1433	2	2.1792
FT 298105	571.9066	465.9806	3	2.1767
FT 147308	435.8366	334.8184	3	2.1739
FT 69089	594.6089	543.7169	3	2.1711
FT 147056	415.6546	528.5205	2	2.1381
FT 267166	441.8816	428.2817	3	2.1268

Table S2: Top 50 features selected for the PLSR of the milk samples. The features listed have the VIP scores.

Feature name	*m*/*z*	rt [s]	charge	VIP score
**FT 196375**	322.9788	230.3010	1	1.6993
**FT 113694**	399.6896	587.1291	2	1.6491
**FT 152646**	472.1981	614.6866	2	1.6319
**FT 100975**	376.1899	587.4633	2	1.6277
**FT 10104**	415.1563	614.5459	2	1.6210
**FT 221952**	620.3291	403.8281	2	1.6130
**FT 34290**	442.1821	320.1580	2	1.5733
**FT 69089**	594.6089	543.7169	3	1.5687
**FT 90590**	502.1869	577.8206	1	1.5678
**FT 53501**	411.0683	278.2279	1	1.5637
**FT 267166**	441.8816	428.2817	3	1.5516
**FT 76342**	677.3094	558.1433	2	1.5499
**FT 191588**	473.9707	330.9609	1	1.5207
**FT 178084**	371.6517	278.3826	2	1.5136
**FT 306791**	361.6671	586.9512	2	1.4969
**FT 128885**	415.2385	538.6355	1	1.4826
**FT 179339**	442.6500	281.1634	2	1.4779
**FT 91496**	495.6959	614.9525	2	1.4715
**FT 49917**	379.1337	538.4349	2	1.4673
**FT 147056**	415.6546	528.5205	2	1.4666
**FT 62883**	857.3572	465.8155	2	1.4530
**FT 242812**	485.1996	324.6662	2	1.4478
**FT 171862**	407.1450	527.7332	2	1.4463
**FT 298105**	571.9066	465.9806	3	1.4404
**FT 33162**	559.7524	431.0715	2	1.4365

Feature name	*m*/*z*	rt [s]	charge	VIP score
**FT 115986**	612.2438	544.2186	3	1.4346
**FT 292662**	474.2066	421.1174	3	1.4279
**FT 147308**	435.8366	334.8184	3	1.4206
**FT 71731**	618.7451	420.4682	2	1.4197
**FT 4584**	291.0472	209.2680	1	1.4053
**FT 274790**	295.4613	320.3514	3	1.3839
**FT 254547**	643.3843	567.6759	1	1.3791
**FT 254413**	792.1684	591.7925	4	1.3710
**FT 191055**	441.7860	363.5643	2	1.3709
**FT 211940**	320.1315	527.7184	2	1.3697
**FT 113301**	649.7499	360.4099	2	1.3684
**FT 109736**	357.1203	538.6249	2	1.3627
**FT 207962**	604.9517	455.1485	3	1.3620
**FT 32164**	479.5760	463.4347	3	1.3611
**FT 185096**	493.7332	378.2085	2	1.3609
**FT 148028**	653.7307	613.2330	2	1.3586
**FT 128773**	439.1532	527.8617	2	1.3538
**FT 49831**	459.8838	454.6533	3	1.3488
**FT 171172**	723.2203	373.0550	3	1.3470
**FT 206290**	292.1198	586.6112	2	1.3448
**FT 43247**	437.1879	803.5245	1	1.3436
**FT 170487**	653.2511	334.2747	2	1.3381
**FT 73029**	433.6436	280.5145	2	1.3377
**FT 281834**	801.2770	538.5818	1	1.3344
**FT 222117**	316.2141	340.5794	1	1.3261
